# Living with osteoporosis: a qualitative descriptive study

**DOI:** 10.1007/s11657-025-01614-4

**Published:** 2025-10-08

**Authors:** Mara Tormen, Chiara Tedesco, Vicente Bernalte-Martì, Angela Cuoco, Anna Maria Carratoni, Gianluca Pucciarelli, Ercole Vellone, Maddalena De Maria, Emanuela Basilici Zannetti, Noemi Cittadini, Annalisa Pennini, Umberto Tarantino, Rosaria Alvaro

**Affiliations:** 1 Department of Biomedicine and Prevention, University of Rome Tor Vergata, Rome, Italy; 2 Universitat Jaume I Facultad de Ciencias de La Salud, Castellón de La Plana, Spain; 3 IRCCS Istituto Ortopedico Rizzoli, Bologna, Italy; 4 Faculty of Nursing and Midwifery, Wroclaw Medical University, Wroclaw, Poland; 5 Faculty of Medicine and Surgery, University “Our Lady of Good Counsel”, Rruga Dritan Hoxha, 1000 Tirana, Albania

**Keywords:** Osteoporosis, Nursing, Perceptions, Emotions, Qualitative research, Chronic illness

## Abstract

***Summary*:**

Osteoporosis affects patients emotionally, yet this aspect is often overlooked. This study found that fear, identity changes, and communication issues shape daily life and self-care. Trust in healthcare providers fosters positive engagement. Addressing emotional experiences can enhance patient-centred osteoporosis care and support treatment adherence.

**Purpose:**

To explore the emotional experiences of individuals living with osteoporosis, with the aim of improving understanding of how these emotions influence self-care behaviours and treatment adherence.

**Methods:**

In this qualitative descriptive study, we conducted in-depth semi-structured interviews with 20 participants diagnosed with osteoporosis, recruited through convenience sampling from an outpatient osteoporosis clinic in central Italy. We coded the interviews deductively and analyzed data using Mayring’s qualitative content analysis framework. We used a priori thematic saturation as the criterion for stopping sampling. We reported data in accordance with the Consolidated Criteria for Reporting Qualitative Studies (COREQ) checklist.

**Results:**

Participants (1 male, 19 females; age range 55–78 years) expressed a wide emotional spectrum associated with living with osteoporosis. Four overarching themes were identified: (1) emotional and psychological impact of the disease, (2) relationship with one’s identity and body, (3) interaction with healthcare professionals, and (4) managing the disease in daily life. Negative emotions, including fear, anxiety, and frustration, were frequently linked to uncertainty about treatment, insufficient communication with healthcare providers, and concerns about the disease progression. Conversely, positive emotions such as trust, hope, and satisfaction emerged when participants felt supported and engaged in their care. These emotions were associated with a stronger motivation for self-management and treatment adherence.

**Conclusion:**

Living with osteoporosis involves a complex interplay of emotional responses that significantly affect patient engagement and disease management. Promoting effective communication and fostering trust between patients and healthcare providers are essential to supporting emotional well-being and enhancing adherence to osteoporosis care plans.

## Introduction

Osteoporosis is a major global health issue characterized by bone mass reduction and micro-architectural deterioration, leading to increased tissue fragility and a higher risk of fractures [1]. More than 200 million people worldwide are currently affected, a number projected to grow substantially with global population ageing [1, 2]. Between 1990 and 2019, osteoporosis-related deaths increased by 111% and disability-related life years lost by 94%, highlighting its rising global burden [3].

Beyond its clinical consequences, osteoporosis has a profound impact on patients’ lives, influencing daily functioning, social interactions, and psychological well-being [4]. Qualitative research is particularly valuable for understanding these personal experiences, as it provides insight into the meaning patients assign to their condition and how these perceptions shape health behaviours [4].

While the physical consequences of osteoporosis, such as fragility fractures, have been extensively studied, less is known about the emotional dimension of living with the disease [4, 5]. 

Previous studies have shown two contrasting perspectives: some patients perceive osteoporosis as a minor condition requiring little attention [4, 6, 7], often underestimating its risks and thereby increasing the likelihood of medication nonadherence [8–10]. Others, by contrast, view it as a highly disruptive illness, associated with isolation, fear of the future, and loss of identity [4, 5]. Other studies have reported uncertainty about risks, treatments, and interactions with healthcare professionals, occasionally framed as opportunities for self-care [4, 5]. Importantly, osteoporosis has been identified as a risk factor for depression [11, 12], poor self-care behaviours and coping strategies [6, 9, 11–16], inequities in healthcare, profound changes in identity [4, 17–20], and low medication adherence, which are essential for improving morbidity and mortality [8].

Literature also reports how fragility fractures significantly impact the overall quality of life, contributing to increased levels of anxiety, altered social interactions, and affected physical appearance and self-image, highlighting that these changes can be as distressing as pain and functional limitations [12, 16, 17]. 

Despite this body of work, studies exploring patients’ lived experiences and the emotional impact of the disease remain limited. A qualitative meta-ethnography by Barker et al. provided a synthesis of patient perspectives but was published in 2016 [4]. A more recent meta-synthesis [21] examined knowledge and concerns regarding bone health; however, its scope was general and did not fully capture the lived experience of osteoporosis nor explore the emotions associated with it in depth.

More recent primary research has provided important insights, yet it largely focuses on specific and isolated aspects of the OP experience, such as knowledge or perceptions of the disease, the diagnosis or the treatments [5–8, 10, 22], patient-reported barriers [9], and physical activity interventions [23]. Additional research has explored lifestyle integration of management advice [24], the impact of physical changes [17], participation in symptom management programmes [25], men’s perceptions [18–20], and quality of life outcomes in older adults [26]. While these contributions are valuable, they remain fragmented and do not capture the broader and holistic lived experience of osteoporosis or the emotional dimensions of living with this condition.

While previous research has highlighted perceptions, beliefs, or aspects of disease management, emotions have often been reported incidentally rather than analyzed comprehensively and globally.

Our study aims to provide an updated and comprehensive overview of the range of emotions associated with osteoporosis but also to offer novel insights into their relative frequency and prominence, thereby highlighting which emotional responses most strongly shape patients’ experiences and self-care.

## Methods

### Design

We conducted a qualitative descriptive study [27] with regular team discussions to minimize bias. We used a non-probabilistic convenience sampling strategy [28, 29] and chose a priori thematic saturation as the criterion for stopping sampling [27, 30, 31]. We conducted a content qualitative analysis following the seven steps of the Mayring method [32] and revised the assessment principles midway through the interviews to check their appropriateness to the research question. After processing all the interviews, we reported the findings narratively, organizing them into relevant themes and incorporating participants’ quotations. We ensured the quality criteria and discussed them following Lincoln and Guba’s criteria [33].

### Inclusion and exclusion criteria

We included patients aged ≥ 65 years for men and ≥ 50 years or postmenopausal for women, with a primary diagnosis of osteoporosis (either senile or postmenopausal), a T-score lower than −2.5 standard deviations (SD) on bone mineral density assessment, cognitive orientation, and provision of informed consent.

Patients were excluded if they were younger than the specified age thresholds, had secondary forms of osteoporosis, exhibited a T-score higher than −2.5 SD, experienced cognitive impairment, faced language barriers, did not provide informed consent, or withdrew from the study. Secondary osteoporosis was excluded through a detailed review of the participants’ medical records. Additionally, women with a history of bilateral oophorectomy (with or without hysterectomy) were excluded due to iatrogenic menopause, whereas those with hysterectomy alone (without removal of the ovaries) were eligible for inclusion.

### Study setting and recruitment

Participants were recruited using a convenience sampling method from the OP Outpatient Clinic at the Policlinico Tor Vergata, a general hospital in central Italy, between March and June 2023 [34]. All eligible patients attending the clinic during the recruitment period were invited to participate.

Clinical and sociodemographic data were collected in person, concurrent with obtaining informed consent. Of the 74 patients approached, 51 declined to participate due to the inability to use technological devices (as interviews were conducted via video call) or a lack of interest. Among the 23 patients who consented, three withdrew during the study due to a loss of interest or challenges in attending the interviews. We approached participants in a welcoming and supportive manner to encourage open and comfortable expression. Once they felt at ease, we collected their sociodemographic data and conducted a semi-structured interview to elicit detailed descriptions of their experiences of living with OP [35]. We stopped convenience sampling after interviewing 20 participants, as data saturation was reached.

### Data collection

Interviews were performed by two trained nursing PhD students via video call (using the WhatsApp VoIP system). Interviews were conducted remotely via video calls using the WhatsApp application, with participants situated in their homes. No individuals other than the participant and the interviewer were present during data collection. A semi-structured interview guide with predefined questions and prompts was used to facilitate comprehensive responses (Appendix Table 3). The first three interviews served as a pilot test to assess the suitability of the set of questions to answer the research question and their comprehensibility and were included in the analysis.

On average, the interviews lasted for an average of 23 min (ranging from 7 to 36 min) and were all audio-recorded and transcribed verbatim by the same interviewers who did the interviews [32]. During the interviews, the interviewers also took field notes to document general impressions and specific characteristics of each participant to inform data analysis. The transcriptions were not returned to the participants; however, key points were summarized during the interviews for clarification and participant input.

### Data analysis

**Fig. 1:**
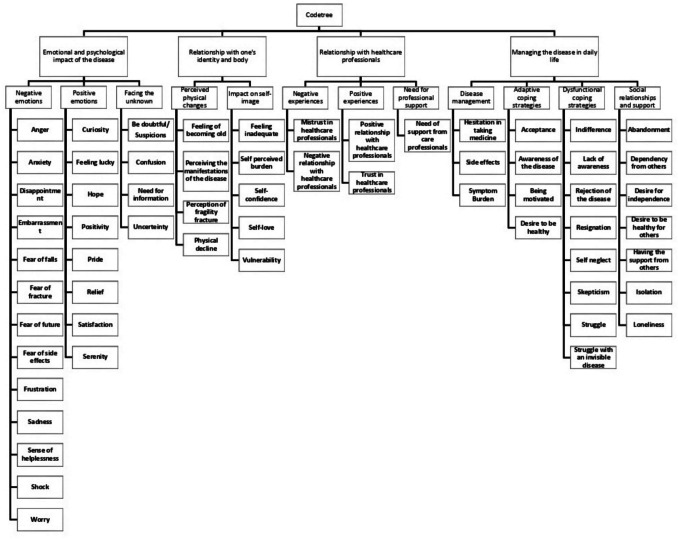
Code tree

We performed a qualitative content analysis following Mayring’s methodology [32]. We developed a codebook deductively, consisting of 62 codes grouped into 12 categories and four general themes. Each code was given a definition derived from previous studies on the topic. Two coders blindly and independently coded the data and were supervised by a third coder who is an expert in qualitative methods. This expert reviewed the analyses and resolved any conflicts between the two coders during the coding process. We applied an *emotion coding* technique to develop codes related to the emotions expressed by participants regarding their experience with OP [36]. After coding 10% of the transcriptions, the coders reviewed the coding guidelines and codebook to ensure alignment with the research question. See the code tree in Fig. 1. The anchor samples and the codebook are available as supplementary material (Appendix  Table 4–5).

### Rigour and reflexivity

The research team had prior experience in qualitative data collection and analysis, particularly in chronic disease contexts, and received specific training through doctoral seminars. The interviewers were knowledgeable in osteoporosis research, having previously been involved in related studies. However, no prior relationship existed between the interviewers and participants. Due to the transient nature of participants in the clinic, ongoing engagement for participant feedback on findings was not feasible. To ensure the credibility of the results, we engaged in prolonged interactions with the participants, while dependability was maintained through a detailed description of data collection and analysis. The involvement of research team members with different areas of expertise strengthened the confirmability of the study. To enhance the transferability of the results, we provided a detailed description of the participants’ experiences [37].

### Ethical consideration

We obtained ethical approval from the Independent Ethics Committee of the Tor Vergata Polyclinic on December 2, 2022, under registration number 211.22. All participants provided informed consent prior to participation. Participants were assured of anonymity and confidentiality; no identifying information was included in the transcripts or results. Each participant was assigned a unique code, followed by age and gender for clarity of interpretation. Audio recordings were securely stored on a password-protected device and were permanently deleted after transcription and data verification.

## Findings

### Sociodemographic and clinical variables

The study participants were 20, aged between 55 and 78 years (mean = 67.25 ± 6.21 SD), all Italian. Most of the sample (65%, *n* = 13) was married or cohabiting and retired (60%, *n* = 12). Of the sample, 60% (*n* = 12) reported no history of fragility fractures (Table 1).

**Table 1:** Sociodemographic and clinical characteristics of participants

ID	Sex	Age	Marital status	Education	Occupation	Year of diagnosis	Accidental falls	Fracture, site	Treatment
I001	Female	68	Single	High school	Retired	2016	Yes		Alendronate; vitamin D
I002	Male	64	Married	Lower secondary school	Retired	2020	No		Denosumab; vitamin D
I003	Female	67	Married	High school	Housewife	2018	Yes		Calcium carbonate + cholecalciferol
I004	Female	59	Widowed	Lower secondary school	Unemployed	20/04/15	Yes		Vitamin D; calcium carbonate; bisphosphonates; rosuvastatin
I005	Female	60	Widowed	High school	Retired	2021	No		No treatment
I006	Female	69	Married	High school	Retired	2013	Yes	Yes, left malleolus	Alendronate + cholecalciferol; vitamin D
I007	Female	75	Single	Elementary school	Retired	2023	Yes	Yes, wirst	Bisphosphonates
I008	Female	70	Married	Lower secondary school	Housewife	2015	No	Yes, vertebral	Denosumab
I009	Female	71	Married	Lower secondary school	Retired	2010	Yes	Yes, vertebral	Alendronate + cholecalciferol; vitamin D
I010	Female	55	Divorced	Lower secondary school	Manual worker	2022	Yes		Bisphosphonates
I011	Female	72	Single	Lower secondary school	Retired	2015	No		Denosumab; vitamin D
I012	Female	76	Married	High school	Retired	2013	Yes	Yes, left malleolus	Denosumab; vitamin D
I013	Female	70	Married	High school	Retired	2013	No	Yes, vertebral	Denosumab; vitamin D
I014	Female	62	Married	High school	Employee	2016	No		Denosumab; vitamin D
I015	Female	64	Married	High school	Unemployed	2017	No		Bisphosphonates; vitamin D
I016	Female	63	Single	University degree	Employee	2008	Yes		Denosumab
I017	Female	60	Married	Lower secondary school	Unemployed	2020	No	Yes, pelvic	Denosumab; vitamin D
I018	Female	78	Married	Elementary school	Retired	2000	Yes	Yes, left and right femurs	Bisphosphonates; vitamin D
I019	Female	73	Married	Lower secondary school	Retired	2013	No		Calcium carbonate + cholecalciferol; vitamin D; denosumab
I020	Female	69	Married	High school	Retired	2013	No		Denosumab

### Overview of coded data

A total of 992 emotion-related codes were identified in the interviews. The average number of codes per interview was 50, with a maximum of 118 and a minimum of 18. The most frequently reported emotions corresponded to the following codes: *being motivated (81 codes)*,* uncertainty (47 codes)*,* confusion (45 codes)*,* need of support from care professionals (44 codes)*, and* disappointment (45 codes).* In the following paragraphs, the most frequent codes for each theme are presented. The total frequency for each code is shown in Table 2, and the code frequencies for each interview are listed in Appendix  Table 6. Frequencies are provided solely as descriptive indicators to enhance transparency and contextualize the distribution of emotional experiences, without implying that a theme’s importance depends on its occurrence.

**Table 2:** Total frequency for each code

Code	Frequency	Code	Frequency
Abandonment	8	Need for information	4
Acceptance	14	Need of support from care professionals	44
Anger	20	Negative relationship with healthcare professionals	37
Anxiety	22	Not perceiving pain	1
Awareness of the disease	1	Perceiving the manifestations of the disease	27
Be doubtful/suspicions	10	Perception of fragility fracture	2
Being motivated	81	Physical decline	10
Confusion	45	Positive relationship with healthcare professionals	25
Curiosity	5	Positivity	30
Dependency from others	4	Pride	2
Desire for independence	8	Rejection of the disease	16
Desire to be healthy	5	Relief	2
Desire to be healthy for others	4	Resignation	17
Disappointment	45	Sadness	8
Embarrassment	-	Satisfaction	20
Fear of falls	15	Self-neglect	34
Fear of fracture	17	Self-perceived burden	12
Fear of future	23	Self-confidence, self-esteem	9
Fear of side effects	7	Self-love	7
Feeling inadequate	5	Sense of helplessness	1
Feeling lucky	26	Serenity	17
Feeling of becoming old	9	Shock	5
Frustration	30	Side effects	15
Having the support from others	1	Skepticism	1
Hesitation in taking medicine	20	Struggle	13
Hope	12	Struggle with an invisible disease	10
Indifference	3	Symptom burden	33
Isolation	1	Trust in healthcare professionals	34
Lack of awareness	1	Uncertainty	47
Loneliness	3	Vulnerability	15
Mistrust in healthcare professionals	26	Worry	23
Total		992	

### Emotional spectrum and key themes

The analysis revealed a diverse emotional spectrum, which was organized into prominent themes: *emotional and psychological impact of the disease*,* relationship with one’s identity and body*, *relationship with healthcare professionals*, and *managing the disease in daily life.*

In general, positive emotions and a trusting relationship with healthcare professionals were more frequent among younger participants (average age 64.4) (“I certainly found some knowledgeable people who, let’s say, definitely gave me reinforcing advice regarding what I was already doing.” Pt. 017, 60 years), while older patients tended to express negative emotions and feelings of distrust or a greater need for support from healthcare professionals (average age 69.5) (“… when they call you for these visits they should also be much clearer…but instead they don’t tell you anything. Honestly, I don’t like it.” Pt. 012, 76 years).

### Emotional and psychological impact of the disease

Participants frequently expressed negative emotions related to their condition, such as anger, anxiety and worry, disappointment, fear, and frustration.

Anger was expressed towards how the disease has been managed and the healthcare system. (“It is true that…if the doctor dedicates 5 more minutes to each patient, I think the patient would be more satisfied because like this it’s zero [emphasizing the word ‘zero’ by raising the voice]. Really, it’s zero!” Pt 012). Participants reported also anxiety and worry about the disease progression, medication side effects, and future health outcomes. (“The side effects…of the medications I take to treat the illness…this creates this anxiety, [sighs]…if I make a mistake, if I do something wrong…This whole situation creates this constant anxiety, so you’re never at ease.” Pt 011). Additionally, for individuals who were informal caregivers of loved ones, OP presented a heightened concern, as they were acutely aware of their responsibility to care for others and the potential consequences of becoming ill themselves.

Disappointment and fear were other negative emotions: the first was related to the relationship with doctors, difficulties in accessing care and the implications of the disease (“Unfortunately, they go to the moon, but they don’t study this other thing [sighs]” Pt 011), the second was related to the future, falls, and fractures that permeate everyday life and influence decisions and behaviours (“I’m always scared, you know? Since I also have the femur at risk of fracture, I’m afraid of falling, you know [sighs].” Pt 010). Participants expressed also frustration which was related to delays in diagnosis or treatment, the side effects of medications, and the limitations imposed by the disease. (“If I had known earlier, I would have started earlier as well, you know.” Pt 010).

Despite the challenges, many participants expressed positive emotions such as positivity, satisfaction, and luckiness. Positivity was related to one’s perspective on life and the impacts that OP has on it (“…In life you can do everything, you just need to be careful, right?” Pt 004). Satisfaction was expressed when participants achieved treatment adherence or physical activity goals. Additionally, patients emphasize how the skills acquired to manage stress, the sharing of the therapeutic journey, and physical activities such as walking on the beach have enhanced their quality of life. Therapeutic assistance was considered essential for both physical and emotional improvement, enabling individuals to approach life with serenity and free from the burden of pain (“In moments of extreme stress, let’s say, I was also able to cope with stress by relying on these skills and knowledge” Pt 014). Additionally, many patients felt lucky because they perceived their condition as less severe compared to others or because they did not experience significant symptoms, emphasizing their perceived fortune in maintaining a good quality of life despite the diagnosis (“There are also those who are worse off” Pt 004).

Patients often expressed a range of emotions related to confronting the unknown, including confusion regarding the disease evolution and the available therapies (“So, I don’t know if it depends on osteoporosis or, as I said before, it could be simple pain or… it’s osteoarthritis, maybe not, maybe it’s not there. But it could be the consequence of osteoporosis, so I don’t know how to link it to that” Pt 002), and a sense of uncertainty related to the prescribed therapies, symptoms, and consequences of OP (“Now they’ve changed my medication, but I’m hesitant—I’m doing some research, investigating.” Pt 001).

### Relationship with one’s identity and body

Participants reported a sense of physical decline related to the effects of the disease and the pain it entails (“I’m practically curling in on myself, I mean I’m bending, hunching over.” Pt 003), along with self-perceived burden (“…having recently lost my husband, and I still have children to think about, I wouldn’t want to be a burden on them, you see, because there’s also this motivation to maintain physical health.” Pt 005). Participants’ perception of the manifestations of the disease highlighted, in some cases, how they were able to notice changes in their health status, while others underscored the fact that OP was often a silent disease and difficult to perceive (“…I don’t feel the OP, but I know it’s there from the graphs and the values, but it’s not something that gives me… I don’t feel anything physically.” Pt 001).

### Relationship with healthcare professionals

The quality of the relationship with healthcare professionals significantly influenced participants’ experiences. Positive relationships with healthcare professionals fostered trust and feelings of support (“My family doctor is always my primary point of reference” Pt 013). However, there were notable expressions of mistrust and reports of negative relationships with healthcare professionals, often tied to insufficient communication or unmet informational needs (“The whole thing, as it was presented to me, also involved the pharmaceutical representative, who seemed to me like a vacuum cleaner salesman [sarcasm]…I interpreted it as – Go ahead and take it because we need to sell it – [laughs]” Pt 001). In general, a strong need for support from care professionals emerged as critical (“Now it has become like the Wild West here, because it’s really not possible to say all these things to the doctors… then they keep changing constantly, and one tells the other, one comes after and the other who arrives knows nothing…” Pt 011).

### Managing the disease in daily life

Participants expressed both positive and negative ways to approach the disease. Those who were more motivated towards self-care were generally younger, and their motivation was accompanied by other positive emotions such as trust in healthcare professionals, positivity, acceptance, and hope. On the contrary, patients who expressed more pronounced self-neglect behaviours were less trusting in their relationships with healthcare professionals, hesitant in taking medications, and displayed feelings of confusion, fear, and frustration with their condition. More specifically, the participants expressed strong self-motivation, self-neglect, hesitation in taking medications, and symptom burden.

Many patients felt a strong self-motivation in taking care of their health because this was an essential personal responsibility for preventing complications and maintaining independence. Their commitment to improving their condition and staying healthy was driven by a desire for longevity, autonomy, and the ability to contribute to the well-being of others, perceiving it not as a burden but as an empowering choice (“Simply taking medication can’t help us… it’s a commitment that the person…takes on, so that the treatment process can have a good outcome and be facilitated” Pt 016). Other patients, on the other hand, expressed a management of their disease driven by self-neglect (“I know what I should do, but I don’t do it, ehh… I’m a bit stupid because by not taking care of myself, I obviously also know what I’m facing, but I don’t try to soften the blow” Pt 001), while many participants, particularly those with a distrustful relationship with doctors, expressed feelings of hesitation in taking medications (“But now they’ve changed my medication, and well, I’m hesitant…I’m doing research, investigations [laughs]” Pt 001). Furthermore, the symptom burden associated with the disease affects numerous participants (“I am practically collapsing in on myself, I mean, I’m bending, hunching over” Pt 003).

## Discussion

### Emotional experiences and perceptions of OP

This study provides valuable insight into the emotional experiences of individuals living with OP, highlighting a complex spectrum of both negative and positive emotions. The novelty of our study lies in its ability to reveal a wide range of emotions, identifying more than 60 distinct codes related to different emotional experiences associated with living with OP. Notably, some of these codes were added ex novo, as they have not been previously reported in the literature, including *Skepticism*,* Self-love*,* Desire to be healthy*,* Indifference*,* Serenity*,* Pride*, and* Relief.*

Our findings are partially consistent with prior research demonstrating that individuals with OP experienced significant emotional distress, characterized by anxiety, uncertainty, fear, and frustration, often linked to disease progression, medication side effects, and the impact of the condition on daily life [4, 5, 11, 12, 21, 26]. The identification of a psychosocial burden of OP, particularly in relation to physical and self-image concerns, fear of fractures, and apprehensions regarding long-term treatment, also confirms the findings of other recent qualitative studies [17, 21, 22].

Moreover, while prior research has highlighted the topic of fear of falls and fractures and the associated limitations [5, 17, 19, 25], our findings interestingly add that this fear is often coupled with a sense of confusion and frustration about the lack of clarity regarding treatment options and a palpable feeling of invisibility within the healthcare system.

Notably, we observed that positive emotions were more prevalent than previously reported [18, 19, 21, 26]. Although this is a qualitative study and no statistical comparisons were performed, there appears to be a descriptive trend suggesting that individuals with fragility fractures may report more negative emotional responses compared with those without fractures, suggesting that perceived disease severity influences emotional responses. This observation should be interpreted cautiously, and further research is needed to explore whether this pattern holds in larger samples.

This explicit focus on emotional valence and variability across clinical subgroups has not been systematically examined in prior research and needs investigation.

### The patient-healthcare professional relationship

Many participants reported feelings of alienation or frustration in their interactions with healthcare professionals, often perceiving neglect or a lack of empathetic communication, which is consistent with findings from previous research [4, 5, 21]. However, our study further identified that these issues were often tied to a lack of trust in the healthcare system, particularly concerning the efficacy of treatments and the long-term management of OP.

A key finding was the direct link between patient trust and emotional well-being. In fact, participants who trusted their healthcare providers were generally more positive, motivated, and satisfied. Conversely, those who lacked trust in healthcare professionals more often expressed negative emotions, such as hesitation regarding medications, self-neglect, confusion, insecurity, fear, anxiety, and disappointment. While individual coping styles may play a role, these findings underscore the critical role of healthcare professionals in shaping patients’ illness experiences and influencing their motivation for treatment adherence, self-care, and consequently the disease outcomes.

### The role of emotions in taking care of one’s health

Our study also explored how emotional responses influence the way individuals take care of their health, such as medication adherence and lifestyle changes. The most frequently coded emotion, *motivation*, was often linked to a strong sense of responsibility for health maintenance, especially among younger participants (59 to 69 years). These individuals were more proactive in managing their condition and expressed more frequently emotions such as positivity, trust, and acceptance of their illness. In contrast, participants who reported higher levels of self-neglect were more likely to report confusion, frustration, anxiety, uncertainty about the treatment, and distrust in healthcare professionals. These results support previous findings which found that a positive perception of one’s health status and treatment options is linked to greater medication adherence [8]. This highlights how taking care of one’s health is not only influenced by rational or knowledge-based factors, suggesting that emotional states and perceptions play a significant role in shaping self-care practices.

### Strengths and limitations

Our study has several limitations. First, the relatively small sample size may not fully capture the diversity of experiences among individuals with osteoporosis, although data saturation was achieved. Second, the use of a convenience sampling method may limit the generalizability of the findings. Although all eligible patients attending the clinic during the study period were invited to participate, the sample may not be representative of the broader population of individuals with osteoporosis. In addition, the sample was recruited from a single outpatient clinic and consisted exclusively of Italian participants, which may restrict the applicability of the results to other geographic or multicultural populations. The gender imbalance observed in the sample may further influence the transferability of the findings. Moreover, although some interviews were relatively brief, they nonetheless yielded meaningful and relevant data that contributed to the thematic analysis. Finally, the reliance on self-reported data may introduce recall and response biases.

Despite its limitations, our study has also several strengths. The adopted qualitative design has offered rich insights into the emotional dimensions of OP. The deductive approach used to code emotional responses was rigorously implemented, following specific methodological criteria and conducted in a blinded manner by multiple researchers. This ensured a structured yet flexible analysis of the interviews, allowing for the identification of a broad spectrum of emotional experiences.

### Recommendations for further research

Future research could build on the findings of this study by examining how emotional responses evolve over time, particularly in relation to fracture events, changes in treatment, or participation in educational interventions. Further investigations could clarify the impact of specific emotions on treatment adherence and self-care behaviours, potentially guiding the development of targeted interventions aimed at improving both emotional well-being and disease management. Finally, cross-cultural studies would be valuable to assess whether the spectrum of emotions identified in this study is consistent across diverse populations.

### Implications for policy and practice

Our findings highlight the importance of integrating emotional support into OP care, moving beyond a purely biomedical approach to a holistic, patient-centred model.

Healthcare providers should be aware of the diverse emotional experiences associated with living with osteoporosis. The identified emotional codes can inform the development of standardized assessment tools and targeted psychoeducational interventions. Incorporating structured emotional assessment and support into routine care may improve patient engagement, adherence, and overall quality of life.

## Conclusion

This study highlights the profound emotional impact of OP, demonstrating its influence on patient perceptions, their engagement in the disease management, and the interactions with healthcare professionals. The spectrum of emotions identified—ranging from anxiety and disappointment to positivity and motivation—demonstrates the dual impact of OP on individuals and underscores the need for a patient-centred approach that acknowledges both physical and psychological aspects of the disease. A particularly noteworthy finding is the profound influence of the patient-healthcare professional relationship on emotional experiences and health management. Trust and effective communication emerged as pivotal factors in shaping patients’ experiences and engagement in care. In contrast, feelings of mistrust and dissatisfaction often coincided with self-neglect, confusion, and reluctance to engage in treatment. By addressing the emotional and relational aspects of care, healthcare professionals can not only improve adherence to treatment but also enhance the overall well-being and quality of life of individuals with OP.

## Data Availability

The data that support the findings of this study are available from the corresponding author upon reasonable request.

## Appendix A

**Table 3:** Main and supportive questions used for the interview

MAIN QUESTION	SUPPORTING QUESTIONS
What does it mean for you to live with osteoporosis?	How long have you been diagnosed?
	What do you know about osteoporosis and its possible complications?
What does "taking care of yourself" mean to you in relation to your osteoporosis?	What are the positive and negative aspects of taking care of your osteoporosis?
	What are your perceptions and attitudes about taking care of your osteoporosis?

## Appendix B

**Table 4:** Anchor samples

Theme	Category	Code	Anchor samples
Emotional and psychological impact of the disease	Negative emotions	**Anger**	- But there should be more check-ups, more, I mean, maybe these visits should be a bit closer together. I do them privately on my own, but as I said, there are many people who can't afford to do so, eh, and people who can’t even afford to buy the medications because for example I’ve also taken supplements, supplements, they’re all paid for, eh! Pt 004. - More than anything, I’m being poorly treated, more than anything. Pt 011. - Now it’s become like a Wild West here, because it’s really not possible to tell all these things to doctors... then they change continuously, one says one thing, the other comes and doesn’t know anything... the one who left (unclear pronunciation), they change all the time. Pt 011 - If you went to the hospital, they’d send you away in the car, and people have died because of this, even for this... and because when they say certain things in (unclear pronunciation), you have to trust them (unclear pronunciation). Then maybe others tell you “it doesn’t matter if there are contraindications.” Eh, not at all! (dialect expression). Well, it’s because of the superficiality of Covid that we couldn’t access treatments, they sent everyone away (sighs). Pt 011. - “No, no, it’s just your impression! It’s just your impression!” The side effect was experienced by almost everyone. Pt 011. - But look, do you think I should now go to the orthopedic specialist and make an appointment? Let’s not talk about it, let’s not talk about it. Pt 012. - Eh... if... let’s say, he washes his hands of it, because it’s already happened to me in other cases, he says “Doctor, I feel like this,” “Eh, well, just go there and they’ll tell you.” Eh... never mind! (dialect expression). Pt 012. - It’s true that the patient, if... if the doctor dedicates 5 more minutes to each patient, I think the patient would be more satisfied because right now it’s zero (emphasizing "zero" loudly). It’s really zero! For me, it’s zero, but for others, it’s not. Alright? Pt 012.
Emotional and psychological impact of the disease	Negative emotions	**Anxiety**	- Because I have problems. Also very serious, immune problems, so. A fracture could bring a normal person a very simple recovery, for me it could lead to other things, other problems with this is. Pt 002. - Then this creates this anxiety, I say (sighs) I try to do it and then I’m anxious so I don’t know... Now. Pt 011. - Eh, I mean.... here it is (sighs), all this situation creates this anxiety situation so you’re never calm. Pt 011. - Then I stopped the therapy because, you know, living with this fear that if you take a treatment, it will make the situation worse, you know... Pt 014. - That one for the spine, it gave me a lot of anxiety (sighs). Pt 015. - This thing gives me a sense of insecurity and instability and makes me feel like this thing is actually there, you know... Pt 017.
Emotional and psychological impact of the disease	Negative emotions	**Disappointment**	- Unfortunately, you go to the moon but you don’t study this other thing (sighs). Pt 011. - (sighs) It’s the pain! The side effects these years give me this general discomfort so I don’t know (sighs)... here I, for example, with the swelling... all those allergies had caused certain things that, look (sighs) had the contraindications... my vision has decreased due to all the negative effects. Pt 011. - When I talk to these [doctors] about osteoporosis, they say - It’s not because of that -. When I talk to the liver specialist, they say, - No, it’s not because of that -. When [laughs] I talk to the lung doctor, they say, - It’s not because of that either - … so, do I have another illness then? [laughs]. Pt 012 -...if it were considered as a disease to prevent, like others, it wouldn’t be bad. I’d feel more followed. Instead, I’m followed by my instinct, my way of being, but not by... not by the healthcare service, you know.Pt 015 - Everything has changed, eh (sighs) because it’s not that it’s here, you have to go at least over 8 kilometers away for the doctor. It’s a fraction here... That’s why I tell you it’s all an adventure sometimes, even if it doesn’t seem like it, but... in fact, here it’s really a dilemma: if someone feels bad and doesn’t move... (sighs) in fact in the area, many times it has happened. Pt 011. - If they treat you from one side, then they create problems from another side. Pt 013. - I always found students. I’ve never (emphasizes the word “never”) had the pleasure of talking to the head of the trial. Pt 013. - You don’t have a, a track, let’s say, of priority, if you’re a certified subject, if you’re suffering from osteoporosis, you don’t have any advantage. Pt 015. - Being a disease that concerns me, millions of women, I imagine, I would dare say... and so it’s a social disease, I mean, I want to say, it has a cost, and all this, so, if it were considered as a disease to be prevented like others, it wouldn’t be bad, I mean, I’d feel more followed. Instead, I’m followed by my instinct, by the way I am, but not by... not by the health service, I mean. Pt 015.
Emotional and psychological impact of the disease	Negative emotions	**Embarrassment**	-/
Emotional and psychological impact of the disease	Negative emotions	**Fear of falls**	- Be careful not to do things that could lead to this consequence. Pt 002. - So, don't tire yourself too much, even the movements you make, be careful... Imagine if you fall, these things... Pt 004. - I’m always afraid, right? Since I also have a femur at risk of fracture, I’m afraid of falling, you know (sighs). So, these things... Pt 010. - But I have to be much more careful because I realize that it might suddenly hurt, and I could lose my balance... okay? Pt 011. - Not now, now it’s terror. So, when I walk on the street, I’m careful, eh, because I’m terrified now, not to say (dialect expression), it’s the truth. Pt 012. - Honestly, this year I was very, very careful: I hadn’t gone for many years. I really like sports: I like skiing, but honestly, I’ve done very, very, very calm things, precisely because, with the awareness that I might fall, or something like that, it would have been difficult for me. Pt 017.
Emotional and psychological impact of the disease	Negative emotions	**Fear of fracture**	- So I always have this fear that in one of my exaggerated movements, I might, I might break something, here. Pt 001. - I could strain myself, I don’t know, fractures, so I avoid making those movements. Pt 008. - I always have the fear, right? Since I also have the femur with fracture risk, I’m afraid of falling, you know (sighs). I mean, these. Pt 010. - I ran to get treated because the idea of a fracture in the spine was unacceptable to me. Pt 014. - I have to be careful; I must not fall. Eh, I must not try in a... because otherwise, maybe I’m at risk of fractures. Pt 019.
Emotional and psychological impact of the disease	Negative emotions	**Fear of future**	- Being careful not to do things that could lead to this consequence, this. Pt 002. - I hope not to end up in a wheelchair. Pt 003. - Of course, I’m worried... I’m worried about the future. Pt 005. - Maybe the attention is also because of this, having seen my mother who, anyway, this disease, it was debilitating for her. Pt 005. - I mean, I’m a bit afraid, here. I repeat, having a husband like this, there’s only me, and I have to try to take care of myself as best as I can, I mean. Pt 010. - It’s always an unknown because it can be a handicap in a certain sense, not a real one, but the beginning of a future handicap; it could be, you know, then it depends on how well it can be treated. Pt 011. - Let’s hope to be able to move forward over time. Pt 020.
Emotional and psychological impact of the disease	Negative emotions	**Fear of side effects**	- What’s... the usual dilemma. I fix one thing, we use these terms, and I have 2 or 3. What’s the point? I then, you know, I also observe... not... (sighs) I see and I asked myself a question that maybe has nothing to do with it; I say: “But why now do I see so many bald women?” And then you find out that they are always the effects of the medicines. I mean, that’s one... it’s one of the effects. Eh, it’s not all... but, I mean. Pt 011. - Then I stopped the therapy because, you know, living with this fear that if you take a treatment... Pt 014.
Emotional and psychological impact of the disease	Negative emotions	**Frustration**	- I think that at least for my case, it would be useful also because I have other health issues. So, one thing is at risk because of the other, that’s the problem. So, for me, it would be important. Pt 002. - Ah, living is... I keep it, what do I have to do? Pt 007. - The fact of having to go out every day, maybe on vacation, and always carry the medication in the cooler bag, that could be a little annoying. Pt 008. - If I had known earlier, I would have started earlier. Pt 010. - I must have been stupid because when I went into menopause at 46, I didn’t do anything about it... I mean, that’s where I think I made a mistake. Pt 010. - I’m too young for... I mean, it gets to me, even when I asked you, do you remember? When I first asked, I saw all these older people, and I said, “Oh my God.” Pt 010. - The negative aspect is this, because I had to get a removable prosthesis instead of being able to do the implant. Pt 013. - These treatments also bring… negative consequences, don’t they? Like that issue with not being able to get the [dental] implant. That was something that… annoyed me a bit, you know. Pt 013
Emotional and psychological impact of the disease	Negative emotions	**Sadness**	- It’s presented this way, unfortunately. Pt 005. - It ruined me. It ruined me. It ruined me in the sense that I couldn’t say anything to anyone anymore. Pt 011.
Emotional and psychological impact of the disease	Negative emotions	**Sense of helplessness**	- Of course, otherwise what do I do? Pt 018.
Emotional and psychological impact of the disease	Negative emotions	**Shock**	- Well, I was a bit traumatized, honestly, I didn’t expect something like this at all. Pt 010. - I saw all these elderly people and thought, "Oh my God." Pt 010. - So at first, it was a bit like I couldn’t recognize myself in this, in this state of... a person who might start having problems. Pt 017.
Emotional and psychological impact of the disease	Negative emotions	**Worry**	- The medications I take to treat the disease. That’s my main problem, you know, and then this creates anxiety, I say (sighs) I try to do it, but then I get anxious, so I don’t know. Pt 011. - I have to do these injections because I have to do them myself, but if I make a mistake, if I do something wrong... (sighs) it’s not like there’s a doctor to call and they come. We used to pay for it before, so someone would come... but now you can only call during that specific time they say, and it’s all complicated... that’s why. Pt 011. - It makes me feel a lot, and it also puts me on guard about the risk of falling, tripping. Pt 017.
Emotional and psychological impact of the disease	Positive emotions	**Curiosity**	- Eh, I study, I study (laughs). I research a lot, in fact, my family doctor told me that I’m a missed doctor (laughs-sighs). So, I mean, I like it, I like the subject, I like to know things, understand. Pt 005. - Yes, it’s a curiosity, more than anything, I mean, I’m fascinated by the world of medicine in general. Pt 005. - I’ve researched a lot, actually, because I also like it, I mean, my profession pushes me to always stay informed. Pt 016.
Emotional and psychological impact of the disease	Positive emotions	**Feeling lucky**	- No, fortunately, until now, I haven’t had any side effects. This is already an important thing. Pt 002. - No, I don’t feel demoralized because I have to take care of myself with this condition. So… - There are others who are worse off. Pt 004. - Rarely, because I’m lucky enough to feel pretty good and haven’t had any major issues in my life. Pt 006. - “Keep going like this”: when I asked him, my father passed away at 89, when I asked him: “Dad, how are you?” his answer was “Keep going.” So it became my way of responding... “How’s it going?” Oh, of course, if I have problems, OK, but if it’s like this, keep going, it’s fine. Pt 006. - Well, it’s not like I’m disabled or missing a limb. I have my legs, and I have my arms too, so I use them. Up to now, let’s say, fortunately, I haven’t had major problems. Pt 014.
Emotional and psychological impact of the disease	Positive emotions	**Hope**	- And let’s hope for the best, that luck will be on our side. Pt 006. - Little by little, I hope something will be found. And, you know, that one will feel a bit of improvement. Pt 007. - I hope they treat me well now, and they won’t do anything else... just look... “go ahead”... like they used to say, “keep going, keep going.” Pt 018. - As long as I feel good, there’s hope... right now I feel fine. I’ll keep going, that’s it. Pt 018. - I hope we have the strength, both physical and mental, to face life at its best. Pt 020.
Emotional and psychological impact of the disease	Positive emotions	**Positivity**	- Well, first of all, don’t get discouraged, so definitely have an attitude... I wouldn’t say "positive," because it’s still a condition, but... tell them that it’s something that, if managed in a certain way or with consistency, is something you can live with peacefully. Pt 017. - Regarding the fact that it’s manageable today... it’s pretty manageable. Pt 017. - I’m the type who doesn’t make a big deal out of things, I always try to overcome everything. Pt 019. - …my father died at 89, when I asked him, – Dad, how are you? – his answer was – Keep going like this! –, so it became my way of answering...Oh, sure, if I have problems, OK, but if it’s like this, keep going, it’s fine. Pt 006. - [OP] it’s something that, if managed in a certain way or with consistency, above all, it’s something you can live with comfortably. Pt 017
Emotional and psychological impact of the disease	Positive emotions	**Pride**	- After I had the surgery, I’m doing well, I manage: I put one foot down, then the other. Pt 018. - Always remarkable compared to my peers who are less... less athletic, so to speak, because I don’t consider myself a particularly sporty person, but I’m definitely someone who moves around and has better mobility than many younger people than me. Pt 020.
Emotional and psychological impact of the disease	Positive emotions	**Relief**	- I also finished the hormone therapy I was on... (clears throat) for cancer because it’s been 10 years, so I don’t take anything. Pt 019. - I’ve found great relief, especially regarding the functional limitation that the pain caused me. Pt 016
Emotional and psychological impact of the disease	Positive emotions	**Satisfaction**	- So, I’m happy to get up in the morning and find myself like I was a few years ago, when I was a bit younger, and to face life with serenity, free from the pain that was lurking there, ready to say "no, you can’t do this, no, you can’t, you’re sick, you need to submit a sick certificate." Pt 016 - Certainly, this person should refer to a center like the one I was lucky enough to find at PTV, because having a single point of contact, getting answers that are always clear and always given with kindness is certainly fundamental. Pt 016 - But all in all, the fact that I don’t have any particular discomforts and I lead a normal life... everything I’ve done over these years makes me think that it has actually helped me manage my life with osteoporosis in a way that is satisfying, all things considered. Pt 017 - I’ve seen a lot of improvement because before there were some things I didn’t want to do because of the exercises, with my hip, I was afraid. But then he said, “No, you shouldn’t be afraid,” he said, “do it, if you feel pain, that’s one thing.” Pt 004 - Instead, with this therapeutic help, I... I’m doing much, much better. Pt 016 - So, we’ve managed this aspect better and I really feel better. Pt 016
Emotional and psychological impact of the disease	Positive emotions	**Serenity**	- In my opinion, we should also be a little more serene (laughs), a bit more serene because this, our way of living has become... I mean, I find it a heavy way, not very reassuring, not very... because it affects, I believe it affects the physical aspect. Pt 005 - I’m calm, you know, I accept what I have and like I said, I take it philosophically. Eh, like they say, you know, that’s all. Pt 007 - Well, it’s just a normal day. I... I manage it however I want, you know. Pt 013 - Well, I can’t really worry about it, by nature, though, eh... Pt 015
Emotional and psychological impact of the disease	Facing the unknown	**Be doubtful/Suspicions**	- A drug can cause problems for heart patients, so I’m trying to investigate a bit to understand. We’ll see. Pt 001. - Having already had four fractures and a vertebral collapse... Actually, I asked myself why the other day they suspended my treatment. Pt 008. - It’s a drug that they say works, but it doesn’t exactly put my bones back in place. But it helps me get along a bit better, right? Pt 010. - Certainly, I always have the doubt whether I’m doing the right thing or doing the wrong thing. Pt 013. - I would like to be sure of the treatment they give me... if it’s the right one, because I don’t know, as I said. Pt 018. - So, the doctors there wanted to give me hormones, but I was quite, how to say... suspicious. Pt 020. - I’ll see the next time in a year, in two years, in three years. Right now, I don’t have any problems, so... No, I don’t know, we’ll see. Pt 020.
Emotional and psychological impact of the disease	Facing the unknown	**Confusion**	- The whole thing, as it was presented to me, then the pharmaceutical representative came, and he seemed like a vacuum cleaner salesman (sarcasm). Maybe they just got the presentation wrong, but I lived it as I read it, like "take it because we have to sell it" (laughs). Pt 001. - So, I don’t know if it’s related to osteoporosis or, as I said before, it could be simple pain, Pt 002. - Oh God, I don’t remember, maybe they told me something, but... I didn’t pay attention… I was just thinking, will I get better? Will I get worse? (laughs) I only thought about that. Pt 007.
Emotional and psychological impact of the disease	Facing the unknown	**Need for information**	- Eh, I study, I study (laughs). I inform myself a lot, in fact my family doctor told me I’m a failed doctor (laughs-sighs). So, I like it, I like the subject, I like to know things, to understand. Yes, maybe I should have gone deeper into it in the past (laughs). Pt 005. - Inserted in that osteoporosis circuit, so this allows me to monitor and keep myself, you know? Updated, in relation to this issue, this criticality. Pt 005. - Eh… no one, I went there to be examined and they showed me this table that was like this and said: “Go and get treated so you can recover.” There you go. Pt 007. - On the other hand, I don’t... what would they be... do you understand that here, I don’t even know what the problems osteoporosis could cause me. Pt 012.
Emotional and psychological impact of the disease	Facing the unknown	**Uncerteinty**	- I don’t know if it’s an effect of osteoporosis or something else, actually. Pt 003 - But is it only that, for sure? Could it be that it’s giving me discomfort? Eh... (sighs) I don’t know... I don’t know why. Pt 012. - On the other hand, I don’t... what would they be... do you understand that here, I don’t even know what problems osteoporosis could cause me. Pt 012. - I can’t tell you if there’s anything that could harm me. I don’t know. Pt 012. - Certainly, I always have the doubt if I’m doing the right thing or the wrong thing. That if you treat one thing and then it breaks somewhere else... Pt 013. - That’s what they told me, because what happened was they gave me too much calcium. Pt 018. - I would like to be sure of the treatment they’re giving me... if it’s the right one, because I don’t know, as I repeat. Pt 018.
Relationship with one's identity and body	Perceived physical changes	**Feeling of becoming old**	- I mean, I saw all these elderly people, I said, “Holy Mary.” Pt 010. - Nothing, because I should start reading (raises the right hand and lets it fall on the leg) to follow all these things. I should read: having osteoporosis, this and that can happen, but I don’t do it, I’m telling you the truth, and even if I do it, I forget about it. Pt 012. - Gradually, with the passing of years, more and more tiredness in the legs. Pt 018. - We’re getting old (laughs), if we’re not already, so... Pt 020.
Relationship with one's identity and body	Perceived physical changes	**Perceiving the manifestations of the disease**	- That I’m practically folding in on myself, I mean, I’m curving, hunching over. Pt 003. - This fact of folding in on myself, and so, objectively, I don’t know if it’s a matter of arthritis or osteoporosis. Pt 003. - So when... sometimes, my back, now I’m feeling better, today it hurts, my hip still hurts a little, but when there’s a bit of pain because osteoporosis, after all, causes pain. Pt 004. - I know myself, and that’s important: recognizing if there’s something wrong. Pt 014. - Because I started having a little bit of limitations. Pt 016
Relationship with one's identity and body	Perceived physical changes	**Perception of fragility fracture**	- I mean, everything related to the break, the fracture. Pt 002. - The only thing I know is that the bones lose their calcium, and therefore you become more sensitive to fractures, even spontaneous ones. Pt 003.
Relationship with one's identity and body	Perceived physical changes	**Physical decline**	- I am practically curling up on myself, I mean, I’m hunching over. Pt 003. - But now I realize that suddenly (sighs) the leg can't hold up anymore, because the pain came to me for the first time with pain in my knee. Pt 011. - So, yeah, I try to do this just to avoid... gaining weight, also because at my age, unfortunately (laughs), metabolism slows down a lot. You need to be careful. Pt 017.
Relationship with one's identity and body	Impact on self-image	**Feeling inadequate**	- A little stupid? A little stupid. Pt 001. - Nothing, nothing, I’m not handling it well (laughs/sarcasm), I’m not handling it well. Pt 001. - I would never make it. I can’t do it because I’m too inconsistent, I try, yeah, I even buy face creams, and then I throw them away after two years because they’ve gotten old, but with the receipt, I just throw them away because I’m inconsistent, unfortunately. I start with good intentions, but I just can’t do it. Pt 001. - I must have been stupid, because when I went into menopause at 46, I never did anything for it, I mean… That’s where I think I made the mistake. Pt 010. - No, no, no, I’m not capable. Also because, in these 10 years, the condition hasn’t really gotten worse, you see? Pt 013.
Relationship with one's identity and body	Impact on self-image	**Self perceived burden**	- No, it doesn’t weigh on me that much, I mean, it’s something that sometimes... can be bothersome. But it doesn’t weigh on me, it can just be annoying. Pt 002. - Yeah, I was telling you, not just in relation to osteoporosis, but I would like to take care of myself in general (laughs). Also because, well, let’s say that, you know... having lost my husband recently, and I still have children to take care of, I don’t want to be a burden to them, you know, because it also triggers this need for physical maintenance. Pt 005.
Relationship with one's identity and body	Impact on self-image	**Self-confidence**	- I trust and rely a lot on my own feelings. Pt 005. - So, well (laughs), eh, actually I keep myself, I’m 62, I think I have a fairly toned body, and I have a pretty clear mind because I’m always studying. Pt 014.
Relationship with one's identity and body	Impact on self-image	**Self-love**	- I love myself, it seems like this term covers a bit of everything. Pt 006. - I find relaxation, and it energizes me psychologically and physically. For example, I have a rather strong awareness of my body. Pt 006.
Relationship with one's identity and body	Impact on self-image	**Vulnerability**	- Because I have some problems, even very serious immune issues, so a fracture that might be a simple recovery for a normal person could lead to other complications for me, other issues. Pt 002. - I’m alone. Pt 004. - Maybe because I’m delicate. Pt 011.
Relationship with healthcare professionals	Negative experiences	**Mistrust in healthcare professionals**	- The whole thing, as it was presented to me, then the pharmaceutical rep arrived, and he seemed like a vacuum cleaner salesman (sarcasm). Maybe they just messed up the presentation, but I experienced it like I read it: “take it because we have to sell it” (laughs). Pt 001. - They didn’t specifically ask me what I have. Pt 001. - I saw a lot of lightness behind it. Pt 001. - There are girls, it’s fine for them to learn, but they should also have a doctor with them who gives, let’s say, the right instructions. Pt 012. - But honestly, they don’t tell you anything, not even how you feel or how you’re doing. Pt 012. - It’s true that if the doctor dedicates 5 more minutes to each patient, I think the patient would be more satisfied because right now, it’s zero (raises voice to emphasize the word “zero”). Really, it’s zero! From my side, it’s zero, but maybe not from others. Fine? Pt 012. - To me, it seems more like an experiment with the Prolia (medicine). To be honest, Pt 013. - He didn’t give me the same treatment. So I don’t know. No... I mean, this, I don’t know why it happens, because I’m doing this treatment, and someone else does another treatment with the same doctor, (sighs) do you understand? I can’t tell you why. Pt 015.
Relationship with healthcare professionals	Negative experiences	**Negative relationship with healthcare professionals**	- In the clinic, they didn’t ask me anything. “Come back in six months, tell us how it’s going, tell us.” But I don’t live inside my bones, so “telling” is a vague thing. What does that mean? So this left me a bit perplexed. Pt 001. - No, no one. I know what the problem is, and as a result, I do it by myself. No one told me anything, that’s it. Pt 002. - Because you see a lot of people… Because, unfortunately, you can’t always get a spot at the hospital, or they don’t insist, right? I insisted a lot to get in there because it’s difficult to get in. It happened that I fell. Pt 004. - Then after a couple of years, they kicked me out because I said (laughs) that I wasn’t paying attention to them. Pt 006. - Eh… no one, they just examined me, and they showed me this chart that was like this, and they said, “Go and get treated, so you’ll recover.” That’s it. Pt 007. - I’ve been treated badly, more than anything, because they didn’t tell me “look, take this, or take that, or there are specialized centers.” I only heard something there, I saw that there are centers... Otherwise, they just said, “Take this,” and you didn’t understand. I wasn’t followed with clear guidance. Pt 011. - Eh, for example, I didn’t know this, because when they call you for these visits, they should also be very clear; they should say, “Look, you’ll take this medicine, and it could cause this. Watch out for these signs.” But they don’t tell you anything. Pt 012. - However, when one goes for a visit, at least... I’m not saying you should stay there for half an hour, but at least (says “at least” slowly) at least they should give some important news. Pt 012. - To me, this Prolia thing feels more like an experiment… I encountered someone who maybe doesn’t even know how this medication works…I’ve never seen the same doctor twice. So, even in that regard, it raises some doubts for me. Pt 013.
Relationship with healthcare professionals	Positive experiences	**Positive relationship with healthcare professionals**	- So, when I have doubts, I feel it, I feel my doctor is very available. Pt 004. - The family doctor with whom I have a good relationship, so I can cover quite a lot, in fact. She, knowing me, knows what kind of person I am, meaning, I’m very curious, right? I’m... so she tries to advise me on what’s best for me, explaining it to me. Pt 005. - I’ve entrusted myself to this professor, who follows me excellently; I have to say, he’s an extraordinary person, and I followed his advice. Pt 016. - The professional, but also the communication and involvement of these extraordinary people helped us a lot, they really guided us in the right direction. They are communicative, truly exceptional professionals. Honestly, it’s really useful to have this kind of professional at our disposal. Pt 016. - Some counseling was also done from this point of view, and I... I followed this advice with pleasure. So, I... I find it favorable, congenial, definitely. Pt 017.
Relationship with healthcare professionals	Positive experiences	**Trust in healthcare professionals**	- They tell me what's wrong, the doctors. Pt 003 - So, in that case, I would immediately consult my doctor. Pt 003 - My general practitioner is an excellent doctor, truly good, I’ve had him since 2007. Pt 004 - I go once a year and follow what they tell me. Pt 006 - Because the only way is to trust the doctors. Pt 008 - Always refer to experts, never do it alone. You don’t have the medical knowledge to give advice... the only ones who can... are the experts. Pt 013 - And at the center, honestly, at PTV, I found this team that I have to say is extraordinary, but why? Because they listen to the person who arrives there with discomfort, with a problem, with pain to manage, which sometimes is a bit complicated. They really welcome you a lot, and it’s extraordinary, extraordinary. Pt 016
Relationship with healthcare professionals	Need for professional support	**Need of support from care professionals**	- It’s true that the patient, if... if the doctor dedicates 5 more minutes to each patient, I think the patient would be more satisfied because otherwise it’s zero (raises voice as she says “zero”). Really, it’s zero! For me, it’s zero, others may not be. Okay? Pt 012 - I always found students. I have never (emphasizes “never”) had the pleasure of talking to the head of the experiment. Pt 013 - I mean, there’s no, let’s say, priority track. If you’re a certified subject, someone with osteoporosis, you get no advantage. Pt 015 - Being a disease that affects me, millions of women, I would say... and so it’s a social disease. I mean, it has a cost, and all this, so if it were considered as a disease to prevent like others, it wouldn’t be bad. I would feel more supported. Instead, I’m guided by my instinct, by the way I am, but not by the healthcare service. Pt 015 - Not having a reference would definitely be an obstacle, definitely an obstacle, but we found one, both my sister and I. Pt 016 - However, when one is being examined, they should dedicate a few more minutes to the person they are examining, but they don’t do it. Pt 012
Managing the disease in daily life	Disease management	**Hesitation in taking medicine**	- Now they’ve prescribed me this new treatment, and I will decide whether to do it or not, and I hope to follow it. Pt 001 - Yes, probably yes, but now they’ve changed the molecule, uh, but I’m hesitant, I’m doing research, investigations. (laughs) Pt 001 - There’s been a sort of push to get people to consume this medication, and that’s one of the reasons for my doubt. Pt 001 - Uh, I don’t do anything (laughs), I don’t immediately take medicine unless it’s an excruciating pain, you know, the kind that is so bad you have to take something. But immediately, if I can resist, I do. Otherwise, at my house, I only have paracetamol, and that’s it (laughs). I use it for headaches or fever, it’s the only medicine I trust. Pt 014 - So, the doctors there wanted to give me hormones, but I was quite, how shall I say... skeptical. Pt 020
Managing the disease in daily life	Disease management	**Side effects**	- Well, for someone who is careful, because with osteoporosis you have some pain and swelling, and you have to be careful in case you fall, you see, especially now for me it has become a bit more pronounced in the hip. Pt 004. - I was feeling cramps, I’m talking about my legs, and they suspended it. Pt 008. - But when you feel too unwell, or, as always, it’s right to do this, but there are different types of effects, and I feel unwell (sighs), for example, I took (sighs) eh I had all this swelling and the fact that I was losing my hair and (sighs) it’s a general malaise (sighs), also because I was feeling unwell with my stomach. Pt 011. - The medicines that are available now, eh (sighs), but for me, it’s always the same problem: finding a middle ground that doesn’t harm everything else. Pt 011.
Managing the disease in daily life	Disease management	**Symptom Burden**	- So, sometimes... my back, now I’m doing better, today it hurts, my hip still hurts a bit, but when there’s some pain, because osteoporosis causes pain anyway. Pt 004 - For example, I had (sighs)... I had all this swelling and the fact that I was losing my hair and (sighs) just a general discomfort (sighs) also because I wasn’t feeling well with my stomach. Pt 011 - I suffer a lot from knee pain. If I squat, I can’t get up. Pt 012 - …little by little, the fatigue kept worsening, and I was limping too, eh... I was limping on the leg... It hurt, it hurt so much, that’s it. Pt 018. - Well, some things I struggle more to do and I don't... I don't walk as much as I used to because I get tired more easily. Pt 019
Managing the disease in daily life	Adaptive coping strategies	**Acceptance**	- So, I adjust, yeah, I adjust. Pt 006 - I’m calm, yeah, I’ll keep what I have and, as I said, I take it like, let’s say, philosophy. Uh, as they say, yeah, that’s all. Pt 007
Managing the disease in daily life	Adaptive coping strategies	**Being motivated**	- If we don’t take care of our health, of our bodies [laughs], I don't see who else should do it! Pt 003. - I don’t think it’s an obstacle to take care of yourself because it means making sure you are as independent as possible, so as not to be a burden to someone. Pt 004. - Uh, it’s good to take care of ourselves, I think; we shouldn’t neglect ourselves. We shouldn’t put symptoms second, right? You might notice something and think: “Ah, I’ll take care of it later,” or “I’ll do something about it,” but we need to be a bit more attentive, yes, that’s true. Pt 005 - I’ve been doing sports twice a week since my daughter was born, and now she’s 37 years old. So, it’s something long-term, I like to walk, and I love the mountains, and I move... I repeat, not obsessively, but still... Pt 006 - So, I make an effort. I try to keep it under control with the MOC, with the treatment I’m doing. Pt 009 - Uh, to have a better life, for me and for those close to me. Pt 009 - I want, I want to take care of myself, I want to (laughs) I really want to take care of myself because I’m too young. Pt 010
Managing the disease in daily life	Adaptive coping strategies	**Desire to be healthy**	- I also try, for personal vanity, not to gain weight (laughs), I try, so, well, I’m careful. I pay attention. Pt 017 - I definitely eat healthy things. Pt 017
Managing the disease in daily life	Dysfunctional coping strategies	**Indifference**	- For me, it’s always the same. Pt 018 - I don’t ask myself questions, oh my, I just have to... I just pay attention when walking, when doing things. Pt 019 - And well, I said, “Well, who cares.” Pt 020
Managing the disease in daily life	Dysfunctional coping strategies	**Lack of awareness**	- Because I know what I should do but I don’t do it... I’m a bit stupid because by not taking care of myself, I obviously know what I’m heading towards, but I don’t try to fend off the blow. Pt 001
Managing the disease in daily life	Dysfunctional coping strategies	**Rejection of the disease**	- I don't take care of it, except for the medication, I don’t do anything else. Pt 001 - It’s not something serious at the moment, so I don’t feel it as a disease. Pt 005 - Maybe it’s a rheumatic problem. Pt 009 - I’m a very active person, so I’m not sedentary, not someone who doesn’t move, who maybe lets their bones atrophy, and so on. Pt 011
Managing the disease in daily life	Dysfunctional coping strategies	**Resignation**	- My laziness, my inconsistency, my being too... I don't honor this temple, which is my body, I don't take care of it. Pt 001 - For me, if I have it, as they say, I’ll keep it. I hope, as I said, I’ll get better taking these pills, that’s it, eh, that's all I hope for. Yes. Pt 007
Managing the disease in daily life	Dysfunctional coping strategies	**Self neglect**	- What does it mean to take care of yourself? I don't know because I've never really taken care of myself, my body, the one that hosts me, I've always mistreated it. Pt 001 - …I’ve never really taken care of myself; my body, the one that hosts me, I’ve always mistreated it. Pt 001. - Because I know what I should do but I don’t do it, ehh... I'm a bit stupid because not taking care of myself, I obviously also know what I’m heading towards, but I don’t try to prevent it. Pt 001 - Nothing, I do nothing, nothing. I take these pills and that’s it, I don’t do anything else, no physiotherapy, nothing, I do nothing. Pt 007. - The exercise, I should do it, but it’s not my thing. I go for a while, and then I don’t go anymore. Pt 012
Managing the disease in daily life	Dysfunctional coping strategies	**Skepticism**	- Oh my God, I have this, if I take this I feel better, I feel worse, no, for me it's always the same. Pt 018
Managing the disease in daily life	Dysfunctional coping strategies	**Struggle**	- It's not easy, really it's not easy, I follow the treatment. Pt 012 - For any movement, any movement, anything... (sighs) anything I like to do, like gardening... eh, (sighs) I have to stop because maybe my back hurts. Pt 013 - Yes, the fact of having fewer opportunities to go out, because also, you know, the ban on... at first, being able to circulate, being able to take walks, it was really heavy, so definitely from a physical limitation point of view and from a psychological point of view because it coincided with a time when I also had to rethink some things regarding the organization of my personal time... it was very negative. Pt 017 - Eh, a little bit, yes, maybe that, because I think it gave problems to everyone to not... eh, I don't know, being stuck like that, without being able to do many things outside, it's a bit annoying, but well... Pt 019
Managing the disease in daily life	Dysfunctional coping strategies	**Struggle with an invisible disease**	- For me, it's difficult to pinpoint, I always have intestinal issues and my stomach always has feelings of nausea, it's been like this for years, so it's not easy for me to understand if it, how to say, affects this because these things come and go for me, both the intestinal problem and the nausea, and now I don’t even know where it's coming from, honestly, maybe from my head (sarcasm). Pt 001 - When I talk to the osteoporotic specialists, they tell me, “it doesn’t depend on this.” When I talk to the liver specialist, they say, “no, it doesn’t depend on this.” When (laughs) I talk to the pulmonologist, they say, “it doesn’t depend on this...” So, am I going to have yet another one? (laughs) Let’s forget it: it’s too much. Pt 012 - No one thinks my disease is related to what I have, according to them... It seems like I have another hidden one. What can I tell you? I don’t know. Pt 012
Managing the disease in daily life	Social relationships and support	**Abandonment**	- It's not like there's a doctor here that you call and they come. Pt 011 - It ruined me. It ruined me. It ruined me in the sense that I couldn’t tell anyone anything anymore. Pt 011 - “No, no, it’s just the impression! It’s the impression!” The side effect almost affected everyone. Pt 011 - You don’t have a, let’s say, priority line, if you’re a certified patient, suffering from osteoporosis, you don’t have any advantage. Pt 015
Managing the disease in daily life	Social relationships and support	**Dependency from others**	- Well, even my husband isn’t doing well (sighs). Pt 012 - I need someone to help me. Pt 012
Managing the disease in daily life	Social relationships and support	**Desire for independence**	- But she does it with me, I don’t think it’s an obstacle, I don’t think it’s an obstacle to take care of oneself, because it’s about making sure you’re as independent as possible, so as not to be a burden to anyone. That’s how I see it, anyway... Pt 004 - At the moment, I do everything by myself (laughs). I... eh, I don’t have anyone behind me in this sense. Pt 005 - Taking care of one’s passions, or your own passions, and... it leads to building self-esteem, which, as you know, is the famous Maslow’s pyramid, right? The personal needs to feel self-fulfilled, and this is the first foundation of being human: if you feel fulfilled and satisfied, you can also face many other situations around you. Pt 014 - I don’t ask for help from anyone, let’s say. Pt 019
Managing the disease in daily life	Social relationships and support	**Desire to be healthy for others**	-I repeat, with a husband like mine, it’s just me, and I have to try to take care of myself as best as I can. Pt 010 -hey need someone who is always there, so I love taking care of myself in this sense, and I’ve undergone screenings and investigations. Pt 016
Managing the disease in daily life	Social relationships and support	**Having the support from others**	-Since she's four years older than me, but for example, she has much more hip problems. But she also keeps it under control; I say, "do this, do that." We talk, you know, she gives me advice, and I give her advice. I think it’s good, right? Pt 004
Managing the disease in daily life	Social relationships and support	**Isolation**	-It ruined me. It ruined me. It ruined me in the sense that I couldn't say anything to anyone anymore. Pt 011
Managing the disease in daily life	Social relationships and support	**Loneliness**	-’m alone, though... Pt 004 -At the moment, I do everything on my own (laughs), I don’t... Eh, I don’t have anyone behind me in that sense. Pt 005 -I repeat, having a husband like this, it’s just me, and I have to try to take care of myself as best as I can. Pt 010

## Appendix C

**Table 5:** Codebook

Theme	Category	Code	Definition	Autor
Emotional and psychological impact of the disease	Negative emotions	**Anger**	Anger about having a disability that choreographs many aspects of life.	Ziebart et al.^4^
Emotional and psychological impact of the disease	Negative emotions	**Anxiety**	Anxiety about every situation that could lead to a fracture.	Barker et al.^2^ Plesh et al.^5^
Emotional and psychological impact of the disease	Negative emotions	**Disappointment**	Sadness or displeasure caused by the non-fulfilment of one's hopes or expectations.	Barker et al.^2^
Emotional and psychological impact of the disease	Negative emotions	**Embarrassment**	OP perceived as a women’s disease. Feeling weak and not masculine.	Barker et al.^2^ Rothmann et al.^3^
Emotional and psychological impact of the disease	Negative emotions	**Fear of falls**	Vigilance about living in a world that is viewed as dangerous. Threatened by normal activities, fear of physical activity. Caution.	Barker et al.^2^ Plesh et al.^5^
Emotional and psychological impact of the disease	Negative emotions	**Fear of fracture**	Deep concern about bones fractures.	Barker et al.^2^ Rothmann et al.^3^
Emotional and psychological impact of the disease	Negative emotions	**Fear of future**	Fear of unpredictable consequences in the future, such as losing mobility, being wheelchair bound, being dependent on others and of further fractures, falls and deformity.	Barker et al.^2^
Emotional and psychological impact of the disease	Negative emotions	**Fear of side effects**	The anxiety of experiencing side effects in taking an anti-osteoporotic treatment.	Rothmann et al.^3^
Emotional and psychological impact of the disease	Negative emotions	**Frustration**	The motivational and/or affective state resulting from being blocked, thwarted, disappointed or defeated.	MeSH
Emotional and psychological impact of the disease	Negative emotions	**Sadness**	Sadness about having a disability that has a profound impact on mobility, work and social lives.	Barker et al.^2^
Emotional and psychological impact of the disease	Negative emotions	**Sense of helplessness**	Learned expectation that one's responses are independent of reward and, hence, do not predict or control the occurrence of rewards. Learned helplessness derives from a history, experimentally induced or naturally occurring, of having received punishment/aversive stimulation regardless of responses made. Such circumstances result in an impaired ability to learn. Used for human or animal populations. [helplessness]	MeSH
Emotional and psychological impact of the disease	Negative emotions	**Shock**	Shocked about the diagnosis of OP. Shocked because the fracture followed an innocuous event.	Barker et al.^2^
Emotional and psychological impact of the disease	Negative emotions	**Worry**	Worrying about doing something wrong, about treatment, about side effects of the OP therapy, about not having enough information.	Rothmann et al.^3^
Emotional and psychological impact of the disease	Positive emotions	**Curiosity**	Exploratory Behavior: The tendency to explore or investigate a novel environment. It is considered a motivation not clearly distinguishable from curiosity.	MeSH
Emotional and psychological impact of the disease	Positive emotions	**Feeling lucky**	Prioritise other health concerns, comparing themselves to people with other “more serious” conditions such as dementia or cancer. OP is a minor health concern.	Barker et al.^2^ Rothmann et al.^3^
Emotional and psychological impact of the disease	Positive emotions	**Hope**	Belief in a positive outcome.	MeSH Barker et al.^2^
Emotional and psychological impact of the disease	Positive emotions	**Positivity**	Positive approach to life, personal resource.	Barker et al.^2^
Emotional and psychological impact of the disease	Positive emotions	**Pride**	A feeling of deep pleasure or satisfaction derived from one's own achievements, the achievements of those with whom one is closely associated, or from qualities or possessions that are widely admired. Some regarded ageing as a natural process, even a time of increased wisdom that brought change and potential benefit.	Barker et al.^2^
Emotional and psychological impact of the disease	Positive emotions	**Relief**	The reduction or removal of pain, distress, or discomfort. It can also mean a feeling of reassurance and relaxation following a period of anxiety or tension.	Graham et al.^12^
Emotional and psychological impact of the disease	Positive emotions	**Satisfaction**	Personal Satisfaction: The individual's experience of a sense of fulfillment of a need or want and the quality or state of being satisfied. Patient Satisfaction: The degree to which the individual regards the health care service or product or the manner in which it is delivered by the provider as useful, effective, or beneficial.	MeSH
Emotional and psychological impact of the disease	Positive emotions	**Serenity**	The state of being calm, peaceful, and untroubled.	Barker et al.^2^
Emotional and psychological impact of the disease	Facing the unknown	**Be doubtful/Suspicions**	Lacking a definite opinion, conviction, or determination. Feeling relates to the complexity and the lack of understanding of the disease.	Barker et al.^2^
Emotional and psychological impact of the disease	Facing the unknown	**Confusion**	Confusion about inconsistent information.	Barker et al.^2^
Emotional and psychological impact of the disease	Facing the unknown	**Need for information**	Patients have unmet information needs about the nature of osteoporosis, medication, self-management and follow-up. Unmet information needs appear to have psychosocial consequences and result in poor treatment adherence.	Raybould et al. ^1^
Emotional and psychological impact of the disease	Facing the unknown	**Uncerteinty**	Uncerteinty about fracture risk, BMD results, the actual benefits of medications	Barker et al.^2^
Relationship with one's identity and body	Perceived physical changes	**Feeling of becoming old**	OP is considered a synonymous of becoming ‘old’. Negative cultural meanings of ageing perceived in a negative meaning, as a personal diminishment, debilitating.	Barker et al.^2^ Rothmann et al.^3^
Relationship with one's identity and body	Perceived physical changes	**Perceiving the manifestations of the disease**	How an individual begins to see himself when living with the revealing evidence of the natural course of osteoporosis	Souza et al.^11^
Relationship with one's identity and body	Perceived physical changes	**Perception of fragility fracture**	Confusion around what is considered a low impact injury, what is a fragility fracture, and what the consequences of the fragility fracture are	Barker et al.^2^
Relationship with one's identity and body	Perceived physical changes	**Physical decline**	The physical manifestation of OP is a reminder of physical decline. OP perceived as an inevitable part of ageing that is beyond personal control.	Barker et al.^2^ Rothmann et al.^3^
Relationship with one's identity and body	Impact on self-image	**Feeling inadequate**	A perceived lack of skills, knowledge, or confidence in managing one's own health, particularly when facing chronic conditions.	Hellqvist et al.^6^
Relationship with one's identity and body	Impact on self-image	**Self perceived burden**	Patient perceptions of the burden that their health conditions exact on family and other caregivers. A multidimensional construct arising from care recipients’ feelings of dependence and guilt at being responsible for the caregiver's hardship.	Tait et al.^15^
Relationship with one's identity and body	Impact on self-image	**Self-confidence**	Individual’s positive self-image.	Rothmann et al.^3^
Relationship with one's identity and body	Impact on self-image	**Self-love**	Self-acceptance and motivation as a means of coping towards developing a new normal.	Tokwe et al.^14^
Relationship with one's identity and body	Impact on self-image	**Vulnerability**	Feeling to be fragile, to be expose to a possible harm.	Barker et al.^2^
Relationship with healthcare professionals	Negative experiences	**Mistrust in healthcare professionals**	Confidence in or reliance on a person or thing (-).	MeSH
Relationship with healthcare professionals	Negative experiences	**Negative relationship with healthcare professionals**	Paternal view of healthcare provision, the healthcare professionals are seen as too busy or as not interested. Women feel to be treated without understanding or knowledge, or to be ignored.	Barker et al.^2^ Rothmann et al.^3^
Relationship with healthcare professionals	Positive experiences	**Positive relationship with healthcare professionals**	A therapeutic relationship incorporated the following: being listened to, being treated with respect, being kept informed and being taken seriously. The women experienced that they were treated with understanding and respect, that health care professionals made an effort to listen, understand and provide support and treatment.	Barker et al.^2^ Rothmann et al.^3^
Relationship with healthcare professionals	Positive experiences	**Trust in healthcare professionals**	Confidence in or reliance on a person or thing.	MeSH
Relationship with healthcare professionals	Need for professional support	**Need of support from care professionals**	Importance of guidance, information and support from care professionals. Important to men’s and women’s insight and their perception of osteoporosis.	Barker et al.^2^ Rothmann et al.^3^
Managing the disease in daily life	Disease management	**Hesitation in taking medicine**	Complex process of deciding whether or not to take medication to manage OP.	Barker et al.^2^ Rothmann et al.^3^
Managing the disease in daily life	Disease management	**Side effects**	Stomach problems to violent nausea, vomiting bile, and burning […] Ways to reduce side effects would be likely to positively influence people’s decisions to remain adherent to these medications.	Salter et al.^16^
Managing the disease in daily life	Disease management	**Symptom Burden**	The subjective, quantifiable prevalence, frequency, and severity of symptoms placing a physiologic burden on patients and producing multiple negative, physical, and emotional patient responses.	MeSH
Managing the disease in daily life	Adaptive coping strategies	**Acceptance**	To accept, among others, a change in the body structure, limitations in physical activity, loss of a social role (withdrawal from professional and family life), chronic fear associated with the risk of bone fracture, anxiety related to chronic and expensive therapy. People who accept certain limitations have a sense of control over their own lives.	Górczewska et al.^13^
Managing the disease in daily life	Adaptive coping strategies	**Awareness of the disease**	The act of taking account of an object or state of affairs. It does not imply assessment of, nor attention to the qualities or nature of the object.	MeSH
Managing the disease in daily life	Adaptive coping strategies	**Being motivated**	Motivation to do physical activity, to stay healthy. Focus on life’s possibilities, on enjoying the possibilities of older adulthood and taking on new challenges. Maintaining meaningful and valued occupations.	Barker et al.^2^ Plesh et al.^5^
Managing the disease in daily life	Adaptive coping strategies	**Desire to be healthy**	Desire to be in good health, maintaining meaningful and valued occupations, a good quality of life and a positive sense of self.	Barker et al.^2^
Managing the disease in daily life	Dysfunctional coping strategies	**Indifference**	Lack of emotion or emotional expression; a disorder of motivation that persists over time. [Apathy]	MeSH
Managing the disease in daily life	Dysfunctional coping strategies	**Lack of awareness**	The condition where an individual does not have sufficient knowledge, understanding, or consciousness about a particular subject, situation, or need.	Tan et al.^9^
Managing the disease in daily life	Dysfunctional coping strategies	**Rejection of the disease**	Feeling they are not the type to get OP, because they have lived a healthy life, or they were protected by physical attributes (strenght, genetics). Refusing OP treatment.	Barker et al.^2^ Rothmann et al.^3^
Managing the disease in daily life	Dysfunctional coping strategies	**Resignation**	A sad feeling of accepting something that you do not like because you cannot easily change it [Cambridge Dictionary]	Lu et al.^7^
Managing the disease in daily life	Dysfunctional coping strategies	**Self neglect**	Profound inattention by individuals to their own health and hygiene.	MeSH
Managing the disease in daily life	Dysfunctional coping strategies	**Skepticism**	Doubt that something is true or useful. [Cambridge Dictionary]	Barcenilla-Wong et al. ^8^
Managing the disease in daily life	Dysfunctional coping strategies	**Struggle**	Have difficulty handling or coping with.	Barker et al.^2^
Managing the disease in daily life	Dysfunctional coping strategies	**Struggle with an invisible disease**	Struggle to accept a diagnosis of OP because they felt healthy and had no visible signs. Struggle to be taken seriously.	Barker et al.^2^ Rothmann et al.^3^
Managing the disease in daily life	Social relationships and support	**Abandonment**	Feeling that no one could help them.	Barker et al.^2^
Managing the disease in daily life	Social relationships and support	**Dependency from others**	The physical manifestation of OP is a reminder of physical decline. OP is perceived as an inevitable part of ageing that is beyond personal control.	Barker et al.^2^
Managing the disease in daily life	Social relationships and support	**Desire for independence**	Desire to maintain personal autonomy and independence, considered fundamental to good health and quality of life.	Barker et al.^2^ Rothmann et al.^3^
Managing the disease in daily life	Social relationships and support	**Desire to be healthy for others**	Spouses and relatives had an important role in relation to being diagnosed with osteoporosis as they were keypersons for support. Importance of being able to help others in order to maintain close relationships.	Rothmann et al.^3^
Managing the disease in daily life	Social relationships and support	**Having the support from others**	Receiving encouragement, assistance, or backing from another person, especially during challenging times.	Rothmann et al.^3^
Managing the disease in daily life	Social relationships and support	**Isolation**	Avoiding social situations, preferring to stay at home in a safe environment.	Barker et al.^2^
Managing the disease in daily life	Social relationships and support	**Loneliness**	Feeling to be alone with the illness.	Barker et al.^2^

## Appendix D

**Table 6:** Codes frequency for each interview

**Pt. 001 – Female – 68 years**	
**Code**	**Frequency**
Be doubtful/Suspicions	1
Confusion	2
Fear of fracture	2
Feeling inadequate	3
Hesitation in taking medicine	5
Lack of awareness	1
Mistrust in healthcare professionals	3
Need of support from care professionals	1
Negative relationship with healthcare professionals	1
Not perceiving pain	1
Perceiving oneself with manifestations of the disease	3
Rejection of the disease	1
Resignation	2
Sadness	1
Self neglect	11
Self perceived burden	1
Struggle with an invisible disease	1
Uncerteinty	2
Vulnerability	1
Worry	1
**Total**	**44**

**Pt. 001 – Female – 68 years**	
**Code**	**Frequency**
Be doubtful/Suspicions	1
Confusion	2
Fear of fracture	2
Feeling inadequate	3
Hesitation in taking medicine	5
Lack of awareness	1
Mistrust in healthcare professionals	3
Need of support from care professionals	1
Negative relationship with healthcare professionals	1
Not perceiving pain	1
Perceiving oneself with manifestations of the disease	3
Rejection of the disease	1
Resignation	2
Sadness	1
Self neglect	11
Self perceived burden	1
Struggle with an invisible disease	1
Uncerteinty	2
Vulnerability	1
Worry	1
**Total**	**44**
**Pt. 002 – Male – 64 years**	
**Code**	**Frequency**
Anger	1
Anxiety	3
Being motivated	4
Confusion	4
Fear of falls	2
Fear of fracture	5
Fear of future	2
Feeling lucky	1
Frustration	1
Need of support from care professionals	1
Negative relationship with healthcare professionals	1
Perceiving oneself with manifestations of the disease	2
Perception of fragility fracture	1
Positivity	2
Self perceived burden	1
Side effects	1
Uncerteinty	4
Vulnerability	2
Worry	3
**Total**	**41**
**Pt 003 – Female – 67 years**	
**Code**	**Frequency**
Being motivated	9
Confusion	5
Fear of falls	1
Fear of fracture	1
Fear of future	3
Feeling lucky	2
Perceiving oneself with manifestations of the disease	3
Perception of fragility fracture	1
Physical decline	2
Positivity	1
Symptom Burden	2
Trust in healthcare professionals	4
Uncerteinty	2
Worry	2
**Total**	**38**
**Pt. 004 – Female – 59 years**	
**Code**	**Frequency**
Anger	2
Being motivated	9
Desire for independence	1
Fear of falls	2
Feeling lucky	3
Having the support from others	1
Hesitation in taking medicine	1
Loneliness	1
Need of support from care professionals	2
Negative relationship with healthcare professionals	1
Perceiving oneself with manifestations of the disease	2
Positive relationship with healthcare professionals	3
Positivity	2
Satisfaction	2
Self perceived burden	2
Side effects	3
Symptom Burden	2
Trust in healthcare professionals	2
Vulnerability	1
Worry	1
**Total**	**42**
**Pt. 005 – Female – 60 years**	
**Code**	**Frequency**
Being motivated	10
Confusion	1
Curiosity	3
Desire for independence	1
Disappointment	1
Fear of future	2
Frustration	1
Loneliness	1
Need for information	2
Need of support from care professionals	1
Negative relationship with healthcare professionals	1
Perceiving oneself with manifestations of the disease	2
Positive relationship with healthcare professionals	1
Positivity	4
Rejection of the disease	1
Resignation	1
Sadness	1
Self perceived burden	1
Self-confidence, self-esteem	2
Serenity	2
Struggle with an invisible disease	2
Uncerteinty	1
**Total**	**42**
**Pt. 006 – Female – 69 years**	
**Code**	**Frequency**
Acceptance	4
Being motivated	8
Confusion	1
Feeling lucky	2
Frustration	1
Hesitation in taking medicine	2
Hope	1
Mistrust in healthcare professionals	1
Negative relationship with healthcare professionals	1
Perceiving oneself with manifestations of the disease	2
Positivity	6
Resignation	1
Self-confidence, self-esteem	1
Self-love	2
Serenity	2
Struggle with an invisible disease	1
Symptom Burden	1
Trust in healthcare professionals	2
Uncerteinty	2
**Total**	**41**
**Pt. 007 – Female – 75 years**	
**Code**	**Frequency**
Acceptance	2
Anxiety	1
Confusion	4
Disappointment	1
Fear of future	2
Feeling lucky	1
Frustration	1
Hope	2
Need for information	1
Need of support from care professionals	2
Negative relationship with healthcare professionals	1
Positivity	1
Rejection of the disease	1
Resignation	3
Self neglect	7
Serenity	1
Symptom Burden	1
Uncerteinty	2
Vulnerability	1
Worry	1
**Total**	**36**
**Pt. 008 – Female – 70 years**	
**Code**	**Frequency**
Anxiety	1
Being motivated	2
Confusion	2
Fear of fracture	3
Fear of future	3
Frustration	1
Hesitation in taking medicine	1
Need of support from care professionals	1
Physical decline	2
Positivity	1
Rejection of the disease	1
Self-love	1
Side effects	1
Trust in healthcare professionals	4
Uncerteinty	1
Vulnerability	3
Worry	1
**Total**	**29**
**Pt. 009 – Female – 71 years**	
**Code**	**Frequency**
Being motivated	3
Confusion	1
Desire for independence	1
Desire to be healthy for others	1
Fear of fracture	1
Perceiving oneself with manifestations of the disease	1
Positive relationship with healthcare professionals	1
Positivity	1
Rejection of the disease	1
Resignation	1
Self neglect	1
Struggle	1
Trust in healthcare professionals	3
Uncerteinty	1
**Total**	**18**
**Pt. 010 – Female – 55 years**	
**Code**	**Frequency**
Be doubtful/Suspicions	1
Being motivated	4
Confusion	1
Desire to be healty for others	1
Fear of falls	3
Fear of fracture	1
Fear of future	2
Feeling inadequate	1
Feeling lucky	2
Feeling of becoming old	1
Frustration	5
Loneliness	1
Mistrust in healthcare professionals	1
Need of support from care professionals	1
Negative relationship with healthcare professionals	1
Perceiving oneself with manifestations of the disease	2
Rejection of the disease	1
Self neglect	3
Self perceived burden	2
Shock	2
Struggle	1
Symptom Burden	3
Trust in healthcare professionals	2
Uncerteinty	2
Vulnerability	1
Worry	2
**Total**	**47**
**Pt. 011 – Female – 72 years**	
**Code**	**Frequency**
Abandonment	5
Acceptance	1
Anger	11
Anxiety	10
Be doubtful/Suspicions	1
Confusion	6
Disappointment	14
Fear of falls	1
Fear of future	4
Fear of side effects	5
Frustration	3
Hesitation in taking medicine	3
Isolation	1
Need of support from care professionals	12
Negative relationship with healthcare professionals	8
Perceiving oneself with manifestations of the disease	1
Physical decline	2
Rejection of the disease	2
Resignation	1
Sadness	4
Self neglect	1
Self perceived burden	1
Shock	1
Side effects	3
Struggle with an invisible disease	2
Symptom Burden	2
Trust in healthcare professionals	1
Uncerteinty	4
Vulnerability	3
Worry	5
**Total**	**118**
**Pt. 012 – Female – 76 years**	
**Code**	**Frequency**
Anger	5
Confusion	6
Dependency from others	2
Disappointment	12
Fear of falls	2
Feeling lucky	1
Feeling of becoming old	1
Frustration	1
Mistrust in healthcare professionals	7
Need for information	1
Need of support from care professionals	14
Negative relationship with healthcare professionals	13
Physical decline	1
Sadness	1
Self neglect	4
Struggle	2
Struggle with an invisible disease	2
Symptom Burden	3
Uncerteinty	6
Vulnerability	1
Worry	2
**Total**	**87**
**Pt. 013 – Female – 70 years**	
**Code**	**Frequency**
Acceptance	2
Be doubtful/Suspicions	3
Being motivated	2
Confusion	1
Disappointment	3
Fear of future	1
Fear of side effects	1
Feeling inadequate	1
Frustration	5
Hesitation in taking medicine	2
Mistrust in healthcare professionals	5
Need of support from care professionals	2
Negative relationship with healthcare professionals	2
Positive relationship with healthcare professionals	4
Sadness	1
Self perceived burden	2
Serenity	2
Side effects	1
Struggle	1
Symptom Burden	1
Trust in healthcare professionals	3
Uncerteinty	2
Worry	1
**Total**	**48**
**Pt. 014 – Female – 62 years**	
**Code**	**Frequency**
Acceptance	1
Anxiety	2
Being motivated	8
Desire for independence	3
Fear of falls	1
Fear of fracture	2
Fear of future	2
Fear of side effects	1
Feeling lucky	2
Frustration	1
Hesitation in taking medicine	3
Perceiving oneself with manifestations of the disease	4
Positive relationship with healthcare professionals	1
Positivity	4
Satisfaction	1
Self-confidence, self-esteem	3
Self-love	2
Side effects	1
Worry	1
**Total**	**43**
**Pt. 015 – Female – 64 years**	
**Code**	**Frequency**
Abandonment	3
Anxiety	4
Be doubtful/Suspicions	1
Being motivated	2
Confusion	5
Disappointment	4
Fear of fracture	1
Fear of future	1
Hope	1
Mistrust in healthcare professionals	1
Need of support from care professionals	3
Negative relationship with healthcare professionals	1
Perceiving oneself with manifestations of the disease	2
Positivity	1
Rejection of the disease	4
Satisfaction	1
Self neglect	2
Serenity	1
Struggle	1
Struggle with an invisible disease	1
Symptom Burden	1
Uncerteinty	2
**TOTALE**	**43**
**Pt. 016 – Female – 63 years**	
**Code**	**Frequency**
Acceptance	1
Being motivated	7
Curiosity	2
Desire for independence	1
Desire to be healthy for others	1
Disappointment	2
Feeling lucky	6
Feeling of becoming old	1
Hope	1
Need of support from care professionals	2
Negative relationship with healthcare professionals	2
Perceiving oneself with manifestations of the disease	1
Positive relationship with healthcare professionals	11
Positivity	1
Satisfaction	10
Self perceived burden	1
Self-confidence, self-esteem	2
Serenity	3
Side effects	2
Struggle	4
Symptom Burden	2
Trust in healthcare professionals	8
**Total**	**71**
**Pt. 017 – Female – 60 years**	
**Code**	**Frequency**
Anxiety	1
Being motivated	7
Desire to be healthy	3
Desire to be healthy for others	2
Disappointment	1
Fear of falls	2
Feeling lucky	2
Feeling of becoming old	1
Frustration	4
Hesitation in taking medicine	1
Perceiving oneself with manifestations of the disease	1
Physical decline	2
Positive relationship with healthcare professionals	4
Positivity	4
Rejection of the disease	1
Satisfaction	3
Self neglect	1
Self-confidence, self-esteem	1
Self-love	1
Serenity	4
Shock	1
Side effects	2
Struggle	2
Symptom Burden	6
Trust in healthcare professionals	5
Uncerteinty	2
Vulnerability	1
Worry	1
**Total**	**66**
**Pt. 018 – Female – 78 years**	
**Code**	**Frequency**
Anger	1
Be doubtful/Suspicions	1
Being motivated	1
Confusion	5
Dependency from others	2
Disappointment	5
Feeling of becoming old	1
Frustration	3
Hope	2
Indifference	1
Mistrust in healthcare professionals	8
Need of support from care professionals	1
Negative relationship with healthcare professionals	3
Pride	1
Rejection of the disease	2
Resignation	7
Satisfaction	2
Self neglect	1
Sense of helplessness	1
Serenity	2
Shock	1
Skepticism	1
Symptom Burden	3
Uncerteinty	10
Worry	2
**Total**	**67**
**Pt. 019 – Female – 73 years**	
**Code**	**Frequency**
Acceptance	1
Awareness of the disease	1
Confusion	2
Desire for independence	1
Disappointment	1
Fear of falls	1
Fear of fracture	1
Feeling lucky	1
Frustration	2
Hope	1
Indifference	1
Need of support from care professionals	1
Negative relationship with healthcare professionals	1
Perceiving oneself with manifestations of the disease	1
Positivity	1
Relief	2
Resignation	1
Self neglect	2
Self perceived burden	1
Struggle	1
Symptom Burden	6
Uncerteinty	2
Vulnerability	1
**Total**	**33**
**Pt. 020 – Female – 69 years**	
**Code**	**Frequency**
Acceptance	2
Be doubtful/Suspicions	2
Being motivated	5
Confusion	1
Desire to be healthy	2
Disappointment	1
Fear of future	1
Feeling lucky	3
Feeling of becoming old	4
Frustration	1
Hesitation in taking medicine	2
Hope	4
Indifference	1
Perceiving oneself with manifestations of the disease	1
Physical decline	1
Positivity	1
Pride	1
Rejection of the disease	1
Satisfaction	1
Self neglect	1
Self-love	1
Side effects	1
Struggle with an invisible disease	1
Uncerteinty	2
**Total**	**41**
